# *N*-(4,5-Diaza­fluoren-9-yl­idene)-4-methyl­aniline

**DOI:** 10.1107/S1600536808039627

**Published:** 2008-12-03

**Authors:** Hui Cang, Dong Jin, Si-Qing Wang, Bin Xu, Jin-Tang Wang

**Affiliations:** a Department of Applied Chemistry, College of Science, Nanjing University of Technology, Nanjing 210009, People’s Republic of China; b College of Chemistry and, Chemical Engineering, Nanjing University of Technology, Nanjing 210009, People’s Republic of China

## Abstract

In the mol­ecule of the title compound, C_18_H_13_N_3_, the 4,5-diaza­fluorenyl­idene unit is nearly planar and is oriented at a dihedral angle of 66.31 (1)° with respect to the benzene ring. In the crystal structure, mol­ecules are stacked regularly along the *c* axis.

## Related literature

For the photochemical properties of 4-methyl-*N*-(4,5-diaza­fluorenyl­idene)benzenamine, see: Wang & Rillema (1997 ▶). For related structures, see: Glagovich *et al.* (2004*a* ▶,*b* ▶); Peters *et al.* (1998 ▶); Wang *et al.* (2006 ▶).
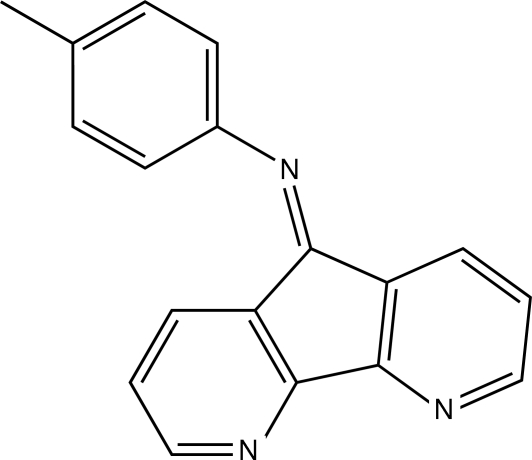


## Experimental

### 

#### Crystal data

C_18_H_13_N_3_
*M* *_r_* = 271.31
Triclinic, 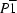

*a* = 7.5970 (15) Å
*b* = 8.6100 (17) Å
*c* = 10.998 (2) Å
α = 77.11 (3)°
β = 87.48 (3)°
γ = 85.79 (3)°
*V* = 699.1 (2) Å^3^
*Z* = 2
Mo *K*α radiation
μ = 0.08 mm^−1^
*T* = 293 (2) K
0.30 × 0.20 × 0.20 mm

#### Data collection

Enraf–Nonius CAD-4 diffractometer
Absorption correction: ψ scan (North *et al.*, 1968 ▶) *T*_min_ = 0.977, *T*_max_ = 0.985
2742 measured reflections
2534 independent reflections
1829 reflections with *I* > 2σ(*I*)
*R*_int_ = 0.025
3 standard reflections every 200 reflections intensity decay: none

#### Refinement

*R*[*F* ^2^ > 2σ(*F* ^2^)] = 0.058
*wR*(*F* ^2^) = 0.167
*S* = 1.00
2534 reflections
190 parameters
H-atom parameters constrained
Δρ_max_ = 0.50 e Å^−3^
Δρ_min_ = −0.28 e Å^−3^

### 

Data collection: *CAD-4 Software* (Enraf–Nonius, 1985 ▶); cell refinement: *CAD-4 Software*; data reduction: *XCAD4* (Harms & Wocadlo, 1995 ▶); program(s) used to solve structure: *SHELXS97* (Sheldrick, 2008 ▶); program(s) used to refine structure: *SHELXL97* (Sheldrick, 2008 ▶); molecular graphics: *SHELXTL* (Sheldrick, 2008 ▶); software used to prepare material for publication: *SHELXTL*.

## Supplementary Material

Crystal structure: contains datablocks I, global. DOI: 10.1107/S1600536808039627/bx2187sup1.cif

Structure factors: contains datablocks I. DOI: 10.1107/S1600536808039627/bx2187Isup2.hkl

Additional supplementary materials:  crystallographic information; 3D view; checkCIF report

## Figures and Tables

**Fig. 1.**
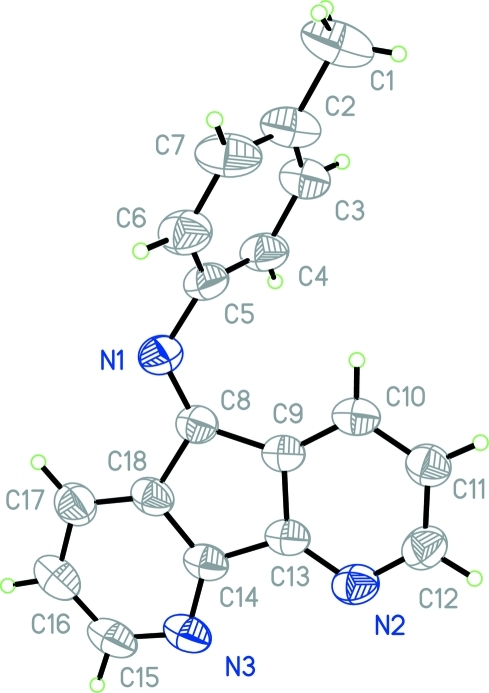
A drawing of the title molecular structure, with the atom-numbering scheme. Displacement ellipsoids are drawn at the 50% probability level.

**Fig. 2.**
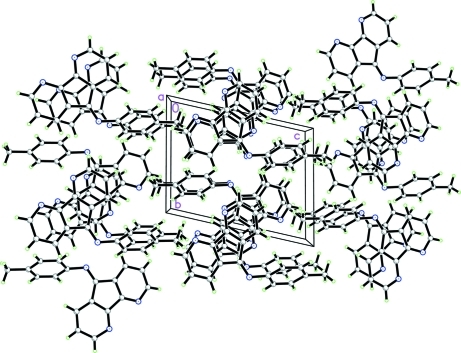
A packing diagram for (I).

## supplementary crystallographic information

### Comment

4-Methyl-*N*-(4,5-diazafluorenylidene)benzenamine, is one of the important ligands, being utilized to synthesize complexes with interesting photochemical properties (Wang & Rillema, 1997). The crystal structure of 4-methyl-*N*-(4,5-diazafluorenylidene)benzenamine monohydrate, (II) (Wang *et al.*, 2006) was reported, previously. We report herein the crystal structure of the title compound, (I), Fig. 1.The bond lengths and angles are comparable with the solvated form (II), and with other fluorenylidene compounds : *N*-fluorenylidene-aniline-benzene (4/1) (III) (Peters *et al.*, 1998), *N*-(9*H*-fluoren-9-ylidene)-*N*-(4-methoxyphenyl)amine, (IV) (Glagovich *et al.*, 2004*a*) and *N*-9*H*-fluoren-9-ylidene-3,4-dimethyl- aniline, (V) (Glagovich *et al.*, 2004*b*). The coplanar ring system is oriented with respect to benzene ring at a dihedral angle of 66.31 (1)°.In the crystal of the title compound, no obvious hydrogen bond is observed, and molecules are stacked regularly along *c* axis, Fig. 2.

### Experimental

The title compound was synthesized by a method reported in literature (Wang & Rillema, 1997). The crystals were obtained by dissolving compound (I) (2.0 g, 6.3 mmol) into solution of acetic ether (50 ml, 1.0 mol/*L*), and evaporating the solvent slowly at room temperature for about 5 d.

### Refinement

H atoms were positioned geometrically, with O—H = 0.82 and C—H = 0.93Å for aromatic H, and constrained to ride on their parent atoms, with *U*_iso_(H) = *xU*_eq_(C/O), where *x* = 1.2 for aromatic H and *x* = 1.5 for other H.

### Crystal data

**Table:**

C_18_H_13_N_3_	*Z* = 2
*M**_r_* = 271.31	*F*(000) = 284
Triclinic, *P*1	*D*_x_ = 1.289 Mg m^−^^3^
Hall symbol: -P 1	Mo *K*α radiation, λ = 0.71073 Å
*a* = 7.5970 (15) Å	Cell parameters from 25 reflections
*b* = 8.6100 (17) Å	θ = 10–13°
*c* = 10.998 (2) Å	µ = 0.08 mm^−^^1^
α = 77.11 (3)°	*T* = 293 K
β = 87.48 (3)°	Plate, yellow
γ = 85.79 (3)°	0.30 × 0.20 × 0.20 mm
*V* = 699.1 (2) Å^3^	

### Data collection

**Table:**

Enraf–Nonius CAD-4 diffractometer	1829 reflections with *I* > 2σ(*I*)
Radiation source: fine-focus sealed tube	*R*_int_ = 0.026
graphite	θ_max_ = 25.3°, θ_min_ = 1.9°
ω/2θ scans	*h* = −9→9
Absorption correction: ψ scan (North *et al.*, 1968)	*k* = −9→10
*T*_min_ = 0.977, *T*_max_ = 0.985	*l* = 0→13
2742 measured reflections	3 standard reflections every 200 reflections
2534 independent reflections	intensity decay: none

### Refinement

**Table:**

Refinement on *F*^2^	Primary atom site location: structure-invariant direct methods
Least-squares matrix: full	Secondary atom site location: difference Fourier map
*R*[*F*^2^ > 2σ(*F*^2^)] = 0.058	Hydrogen site location: inferred from neighbouring sites
*wR*(*F*^2^) = 0.167	H-atom parameters constrained
*S* = 1.00	*w* = 1/[σ^2^(*F*_o_^2^) + (0.05*P*)^2^ + 0.85*P*] where *P* = (*F*_o_^2^ + 2*F*_c_^2^)/3
2534 reflections	(Δ/σ)_max_ < 0.001
190 parameters	Δρ_max_ = 0.50 e Å^−^^3^
0 restraints	Δρ_min_ = −0.28 e Å^−^^3^

### Special details

**Table:**

Geometry. All e.s.d.'s (except the e.s.d. in the dihedral angle between two l.s. planes) are estimated using the full covariance matrix. The cell e.s.d.'s are taken into account individually in the estimation of e.s.d.'s in distances, angles and torsion angles; correlations between e.s.d.'s in cell parameters are only used when they are defined by crystal symmetry. An approximate (isotropic) treatment of cell e.s.d.'s is used for estimating e.s.d.'s involving l.s. planes.
Refinement. Refinement of *F*^2^ against ALL reflections. The weighted *R*-factor *wR* and goodness of fit *S* are based on *F*^2^, conventional *R*-factors *R* are based on *F*, with *F* set to zero for negative *F*^2^. The threshold expression of *F*^2^ > σ(*F*^2^) is used only for calculating *R*-factors(gt) *etc*. and is not relevant to the choice of reflections for refinement. *R*-factors based on *F*^2^ are statistically about twice as large as those based on *F*, and *R*- factors based on ALL data will be even larger.

### Fractional atomic coordinates and isotropic or equivalent isotropic displacement parameters (Å^2^)

**Table:**

	*x*	*y*	*z*	*U*_iso_*/*U*_eq_	
N1	0.2577 (3)	1.3579 (3)	0.5598 (2)	0.0539 (7)	
C1	0.0220 (6)	1.2134 (6)	1.0717 (3)	0.0953 (14)	
H1B	−0.0898	1.2705	1.0794	0.143*	
H1C	0.1063	1.2457	1.1223	0.143*	
H1D	0.0091	1.1008	1.0992	0.143*	
N2	0.2970 (3)	0.8734 (3)	0.3984 (2)	0.0507 (6)	
C2	0.0855 (5)	1.2499 (5)	0.9378 (3)	0.0658 (9)	
N3	0.4535 (3)	1.1454 (3)	0.2071 (2)	0.0526 (6)	
C3	0.2450 (4)	1.1836 (4)	0.8993 (3)	0.0584 (8)	
H3B	0.3152	1.1156	0.9583	0.070*	
C4	0.3018 (4)	1.2158 (4)	0.7759 (3)	0.0551 (8)	
H4A	0.4094	1.1703	0.7528	0.066*	
C5	0.1987 (4)	1.3158 (3)	0.6866 (3)	0.0501 (7)	
C6	0.0411 (4)	1.3877 (4)	0.7236 (3)	0.0602 (8)	
H6A	−0.0268	1.4590	0.6650	0.072*	
C7	−0.0137 (5)	1.3531 (5)	0.8469 (3)	0.0723 (10)	
H7A	−0.1203	1.4002	0.8701	0.087*	
C8	0.2867 (4)	1.2521 (3)	0.4951 (2)	0.0432 (6)	
C9	0.2518 (3)	1.0803 (3)	0.5183 (2)	0.0398 (6)	
C10	0.1712 (4)	0.9757 (3)	0.6156 (3)	0.0463 (7)	
H10A	0.1279	1.0086	0.6868	0.056*	
C11	0.1576 (4)	0.8219 (4)	0.6030 (3)	0.0490 (7)	
H11A	0.1044	0.7485	0.6663	0.059*	
C12	0.2236 (4)	0.7763 (4)	0.4952 (3)	0.0537 (8)	
H12A	0.2157	0.6705	0.4907	0.064*	
C13	0.3085 (3)	1.0227 (3)	0.4114 (2)	0.0421 (6)	
C14	0.3829 (3)	1.1547 (3)	0.3185 (2)	0.0429 (6)	
C15	0.5163 (4)	1.2817 (4)	0.1427 (3)	0.0586 (8)	
H15A	0.5670	1.2820	0.0641	0.070*	
C16	0.5114 (4)	1.4213 (4)	0.1839 (3)	0.0617 (9)	
H16A	0.5590	1.5111	0.1338	0.074*	
C17	0.4364 (4)	1.4296 (4)	0.2994 (3)	0.0545 (8)	
H17A	0.4317	1.5228	0.3292	0.065*	
C18	0.3691 (4)	1.2917 (3)	0.3673 (2)	0.0457 (7)	

### Atomic displacement parameters (Å^2^)

**Table:**

	*U*^11^	*U*^22^	*U*^33^	*U*^12^	*U*^13^	*U*^23^
N1	0.0653 (17)	0.0495 (14)	0.0480 (14)	0.0060 (12)	0.0035 (12)	−0.0170 (11)
C1	0.086 (3)	0.149 (4)	0.054 (2)	−0.014 (3)	0.016 (2)	−0.029 (2)
N2	0.0502 (15)	0.0541 (15)	0.0501 (14)	0.0092 (11)	−0.0023 (11)	−0.0200 (12)
C2	0.058 (2)	0.097 (3)	0.0483 (18)	−0.0093 (18)	0.0060 (16)	−0.0284 (18)
N3	0.0500 (15)	0.0690 (17)	0.0361 (13)	0.0112 (12)	−0.0010 (11)	−0.0111 (12)
C3	0.059 (2)	0.072 (2)	0.0459 (17)	0.0002 (16)	−0.0022 (14)	−0.0174 (15)
C4	0.0563 (19)	0.0615 (19)	0.0507 (18)	0.0054 (15)	0.0036 (14)	−0.0233 (15)
C5	0.0569 (18)	0.0489 (16)	0.0487 (17)	0.0000 (13)	0.0049 (14)	−0.0220 (13)
C6	0.0562 (19)	0.068 (2)	0.057 (2)	0.0105 (15)	0.0007 (15)	−0.0220 (16)
C7	0.055 (2)	0.106 (3)	0.060 (2)	0.0086 (19)	0.0085 (17)	−0.036 (2)
C8	0.0416 (15)	0.0473 (15)	0.0401 (15)	0.0086 (12)	−0.0030 (12)	−0.0117 (12)
C9	0.0340 (14)	0.0480 (15)	0.0382 (14)	0.0065 (11)	−0.0050 (11)	−0.0135 (12)
C10	0.0424 (15)	0.0585 (18)	0.0401 (15)	0.0051 (13)	−0.0046 (12)	−0.0171 (13)
C11	0.0469 (17)	0.0540 (18)	0.0452 (16)	0.0011 (13)	−0.0054 (13)	−0.0096 (13)
C12	0.0573 (19)	0.0476 (17)	0.0589 (19)	0.0029 (14)	−0.0046 (15)	−0.0186 (15)
C13	0.0368 (15)	0.0523 (16)	0.0375 (14)	0.0120 (12)	−0.0069 (11)	−0.0142 (12)
C14	0.0348 (14)	0.0567 (17)	0.0363 (14)	0.0108 (12)	−0.0068 (11)	−0.0116 (12)
C15	0.0571 (19)	0.081 (2)	0.0335 (15)	0.0107 (17)	0.0004 (13)	−0.0087 (15)
C16	0.062 (2)	0.074 (2)	0.0424 (17)	0.0018 (16)	−0.0010 (15)	−0.0003 (15)
C17	0.0590 (19)	0.0542 (18)	0.0468 (17)	0.0046 (14)	−0.0015 (14)	−0.0062 (14)
C18	0.0436 (16)	0.0542 (17)	0.0366 (14)	0.0103 (12)	−0.0056 (12)	−0.0078 (12)

### Geometric parameters (Å, °)

**Table:**

N1—C8	1.277 (3)	C7—H7A	0.9300
N1—C5	1.422 (4)	C8—C9	1.486 (4)
C1—C2	1.501 (5)	C8—C18	1.491 (4)
C1—H1B	0.9600	C9—C10	1.387 (4)
C1—H1C	0.9600	C9—C13	1.414 (4)
C1—H1D	0.9600	C10—C11	1.373 (4)
N2—C12	1.327 (4)	C10—H10A	0.9300
N2—C13	1.334 (4)	C11—C12	1.392 (4)
C2—C7	1.388 (5)	C11—H11A	0.9300
C2—C3	1.391 (5)	C12—H12A	0.9300
N3—C14	1.332 (3)	C13—C14	1.477 (4)
N3—C15	1.337 (4)	C14—C18	1.397 (4)
C3—C4	1.380 (4)	C15—C16	1.374 (5)
C3—H3B	0.9300	C15—H15A	0.9300
C4—C5	1.382 (4)	C16—C17	1.383 (4)
C4—H4A	0.9300	C16—H16A	0.9300
C5—C6	1.394 (4)	C17—C18	1.375 (4)
C6—C7	1.374 (4)	C17—H17A	0.9300
C6—H6A	0.9300		
			
C8—N1—C5	121.0 (3)	C10—C9—C13	117.7 (3)
C2—C1—H1B	109.5	C10—C9—C8	133.8 (2)
C2—C1—H1C	109.5	C13—C9—C8	108.3 (2)
H1B—C1—H1C	109.5	C11—C10—C9	117.7 (3)
C2—C1—H1D	109.5	C11—C10—H10A	121.2
H1B—C1—H1D	109.5	C9—C10—H10A	121.2
H1C—C1—H1D	109.5	C10—C11—C12	119.8 (3)
C12—N2—C13	115.1 (2)	C10—C11—H11A	120.1
C7—C2—C3	117.2 (3)	C12—C11—H11A	120.1
C7—C2—C1	120.8 (3)	N2—C12—C11	124.6 (3)
C3—C2—C1	122.0 (4)	N2—C12—H12A	117.7
C14—N3—C15	114.0 (3)	C11—C12—H12A	117.7
C4—C3—C2	121.8 (3)	N2—C13—C9	125.1 (3)
C4—C3—H3B	119.1	N2—C13—C14	126.4 (2)
C2—C3—H3B	119.1	C9—C13—C14	108.5 (2)
C3—C4—C5	119.9 (3)	N3—C14—C18	125.3 (3)
C3—C4—H4A	120.0	N3—C14—C13	126.1 (3)
C5—C4—H4A	120.0	C18—C14—C13	108.6 (2)
C4—C5—C6	119.2 (3)	N3—C15—C16	124.8 (3)
C4—C5—N1	121.3 (3)	N3—C15—H15A	117.6
C6—C5—N1	119.3 (3)	C16—C15—H15A	117.6
C7—C6—C5	119.9 (3)	C15—C16—C17	120.6 (3)
C7—C6—H6A	120.1	C15—C16—H16A	119.7
C5—C6—H6A	120.1	C17—C16—H16A	119.7
C6—C7—C2	121.9 (3)	C18—C17—C16	115.9 (3)
C6—C7—H7A	119.1	C18—C17—H17A	122.0
C2—C7—H7A	119.1	C16—C17—H17A	122.0
N1—C8—C9	133.1 (3)	C17—C18—C14	119.4 (3)
N1—C8—C18	121.3 (3)	C17—C18—C8	131.6 (3)
C9—C8—C18	105.6 (2)	C14—C18—C8	108.9 (2)
			
C7—C2—C3—C4	−1.2 (5)	C12—N2—C13—C14	−179.6 (3)
C1—C2—C3—C4	179.2 (3)	C10—C9—C13—N2	−2.8 (4)
C2—C3—C4—C5	−0.4 (5)	C8—C9—C13—N2	−179.6 (2)
C3—C4—C5—C6	2.5 (5)	C10—C9—C13—C14	177.7 (2)
C3—C4—C5—N1	176.7 (3)	C8—C9—C13—C14	0.8 (3)
C8—N1—C5—C4	63.1 (4)	C15—N3—C14—C18	1.2 (4)
C8—N1—C5—C6	−122.7 (3)	C15—N3—C14—C13	−178.0 (3)
C4—C5—C6—C7	−2.9 (5)	N2—C13—C14—N3	−1.2 (4)
N1—C5—C6—C7	−177.3 (3)	C9—C13—C14—N3	178.4 (2)
C5—C6—C7—C2	1.3 (6)	N2—C13—C14—C18	179.5 (3)
C3—C2—C7—C6	0.7 (6)	C9—C13—C14—C18	−1.0 (3)
C1—C2—C7—C6	−179.7 (4)	C14—N3—C15—C16	0.0 (4)
C5—N1—C8—C9	8.7 (5)	N3—C15—C16—C17	−0.6 (5)
C5—N1—C8—C18	−172.5 (3)	C15—C16—C17—C18	−0.1 (4)
N1—C8—C9—C10	2.4 (5)	C16—C17—C18—C14	1.2 (4)
C18—C8—C9—C10	−176.5 (3)	C16—C17—C18—C8	177.0 (3)
N1—C8—C9—C13	178.6 (3)	N3—C14—C18—C17	−1.9 (4)
C18—C8—C9—C13	−0.3 (3)	C13—C14—C18—C17	177.4 (2)
C13—C9—C10—C11	2.2 (4)	N3—C14—C18—C8	−178.6 (2)
C8—C9—C10—C11	178.1 (3)	C13—C14—C18—C8	0.7 (3)
C9—C10—C11—C12	0.0 (4)	N1—C8—C18—C17	4.5 (5)
C13—N2—C12—C11	1.5 (4)	C9—C8—C18—C17	−176.4 (3)
C10—C11—C12—N2	−2.0 (5)	N1—C8—C18—C14	−179.3 (3)
C12—N2—C13—C9	0.9 (4)	C9—C8—C18—C14	−0.3 (3)

## References

[bb1] Enraf–Nonius (1985). *CAD-4 Software* Enraf–Nonius, Delft, The Netherlands.

[bb2] Glagovich, N., Reed, E., Crundwell, G., Updegraff, J. B. III, Zeller, M. & Hunter, A. D. (2004*a*). *Acta Cryst.* E**60**, o623–o625.

[bb3] Glagovich, N. M., Reed, E. M., Crundwell, G., Updegraff, J. B. III, Zeller, M. & Hunter, A. D. (2004*b*). *Acta Cryst.* E**60**, o1269–o1270.

[bb4] Harms, K. & Wocadlo, S. (1995). *XCAD4* University of Marburg, Germany.

[bb5] North, A. C. T., Phillips, D. C. & Mathews, F. S. (1968). *Acta Cryst.* A**24**, 351–359.

[bb6] Peters, K., Peters, E. M. & Quast, H. (1998). *Z. Kristallogr. New Cryst. Struct.* **213**, 607–608.

[bb7] Sheldrick, G. M. (2008). *Acta Cryst.* A**64**, 112–122. 10.1107/S0108767307043930 18156677

[bb8] Wang, Y. X. & Rillema, D. P. (1997). *Tetrahedron*, **37**, 12377–12390.

[bb9] Wang, C. X., Wang, P. & Li, Z. F. (2006). *Z. Kristallogr. New Cryst. Struct.* **221**, 211–212.

